# Fabrication of La_2_O_3_ Uniformly Doped Mo Nanopowders by Solution Combustion Synthesis Followed by Reduction under Hydrogen

**DOI:** 10.3390/ma11122385

**Published:** 2018-11-27

**Authors:** Siyong Gu, Mingli Qin, Houan Zhang, Jidong Ma

**Affiliations:** 1 Institute for Advanced Materials and Technology, University of Science and Technology Beijing, Beijing 100083, China; 2 Fujian Collaborative innovation center for R&D of coach and special vehicle, Xiamen University of Technology, Xiamen 361024, China; ha_zhang@163.com (H.Z.); jidongustb@126.com (J.M.)

**Keywords:** Mo nanopowder, doping, La_2_O_3_, solution combustion synthesis, hydrogen reduction

## Abstract

This work reports the preparation of La_2_O_3_ uniformly doped Mo nanopowders with the particle sizes of 40–70 nm by solution combustion synthesis and subsequent hydrogen reduction (SCSHR). To reach this aim, the foam-like MoO_2_ precursors (20–40 nm in size) with different amounts of La_2_O_3_ were first synthesized by a solution combustion synthesis method. Next, these precursors were used to prepare La_2_O_3_ doped Mo nanopowders through hydrogen reduction. Thus, the content of La_2_O_3_ used for doping can be accurately controlled via the SCSHR route to obtain the desired loading degree. The successful doping of La_2_O_3_ into Mo nanopowders with uniform distribution were proved by X-ray photon spectroscopy and transmission electron microscopy. The preservation of the original morphology and size of the MoO_2_ precursor by the La_2_O_3_ doped Mo nanopowders was attributed to the pseudomorphic transport mechanism occurring at 600 °C. As shown by X-ray diffraction, the formation of Mo_2_C impurity, which usually occurs in the direct H_2_ reduction process, can be avoided by using the Ar calcination-H_2_ reduction process, when residual carbon is removed by the carbothermal reaction during Ar calcination at 500 °C.

## 1. Introduction

Molybdenum (Mo) and its alloys have excellent high temperature performance, such as high temperature strength, high creep resistance, low thermal expansion coefficient, and high thermal conductivity [1,2,3]. Thus, they have been widely used as high-temperature components in the aerospace industry, the nuclear industry, metal processing, and other fields [3,4,5]. However, the major drawbacks related to the inherent brittleness and high ductile-brittle transition temperature (DBTT) of Mo alloys not only lead to poor toughness at room temperature, but also to inadequate ductility and strength at high temperature, which greatly limits the widespread application of Mo and its alloys. Therefore, decreasing the DBTT to simultaneously improve both ductility and strength of Mo alloy at room temperature has become a well-known major goal over recent decades [5,6]. As previously reported [2,7,8,9], the addition of rare-earth oxides, such as La_2_O_3_, Y_2_O_3_, and Gd_2_O_3_ into Mo alloys via various doping routes to form rare-earth oxide doped Mo alloys can obviously decrease DBTT. Thus, the rare-earth oxide doped Mo alloys have high toughness and strength at both room and high temperatures. Moreover, the preparation of nano-sized oxide particles with uniform dispersion into the Mo grain and the engineering of Mo grains at nanoscale are key factors for obtaining high performance Mo alloys. Recently, it was clearly demonstrated that Mo alloys can be achieved using high quality La_2_O_3_ doped Mo powder synthesized via the liquid–liquid doping route (L-L doping) [2]. Therefore, the rare-earth oxide doped powders composed of nanoparticles with a highly dispersed oxide phase are a crucial factor to obtain high-performance Mo alloys. So far, the conventional doping routes, i.e., solid Mo powders mixed with solid oxide particles (S-S doping) [7,10], solid Mo powders mixed with liquid oxide particles (S-L doping) [11,12,13], and liquid Mo-based compounds mixed with liquid oxide particles (L-L doping) [2,14], have been applied to prepare the designed rare-earth doped Mo powders. Among these strategies, the L-L doping route is the most attractive because it allows mixing of the precursors at the molecular level. 

As previously reported [15], the performance of La_2_O_3_ doped Mo alloys exceeds those of other doped by rare-earth oxides (i.e., Nd_2_O_3_, Sm_2_O_3_, Gd_2_O_3_, and Y_2_O_3_). In addition, previous studies about the doping content of La_2_O_3_ indicated that the doping content of La_2_O_3_ exceeds 2.0 wt.%, which cannot improve the properties of molybdenum alloys [2,16]. Cheng et al. [8] studied the properties of Mo alloy with different doping contents of La_2_O_3_, and the results showed that the Mo alloy with 0.5–1.0 wt.% La_2_O_3_ doping simultaneously achieve high strength and great elongation.

Solution combustion synthesis (SCS) is a versatile, energy-efficient, and mass production suitable method that has been used to fabricate several types of nano-sized oxides with different physical and chemical properties [17,18,19]. In addition, SCS is also one of the proper methods to prepare uniformly doped nano-sized oxides, such as Al-doped ZnO nanoparticles [20], Sn-doped α-Fe_2_O_3_ nanoparticles [21], and Fe^3+^-W_18_O_49_ nanoparticles [22], because precursor mixing at the molecular level ensured that the doped element can be distributed homogeneously throughout the matrix.

In our previous work [23], foam-like MoO_2_ nanoparticles, assembled by 20–30 nm nanoparticles, were successfully fabricated by SCS. In this paper, a facile method based on the SCS and subsequent hydrogen reduction route (SCSHR) is proposed to prepare La_2_O_3_ uniformly doped Mo nanopowders with various La_2_O_3_ loading degrees ranging from 0 to 1.2 wt.%. In fact, the SCSHR route not only ensures the high dispersion of La_2_O_3_ as a result of mixing at the molecular level, but also the formation of nanopowders with homogeneous distribution of the oxide nanoparticles. The crystalline phase evolution, morphology, elemental composition, and reduction processes have been studied in detail. Moreover, the formation mechanism of La_2_O_3_ doped Mo nanopowders has been discussed.

## 2. Experimental

### 2.1. Materials

Hexaammonium molybdate ((NH_4_)_6_Mo_7_O_24_·4H_2_O, AHM, Sinopharm Chemical Reagent Co., Ltd., Shanghai, China), lanthanum(III) nitrate hexahydrate (La(NO_3_)_3_·6H_2_O, Sinopharm Chemical Reagent Co., Ltd.), ammonium nitrate ((NH_4_)NO_3_, Sinopharm Chemical Reagent Co., Ltd.), and glycine (C_2_H_5_NO_2_, Sinopharm Chemical Reagent Co., Ltd.) were of analytical grade and used as received without any further purification.

### 2.2. Synthesis of La_2_O_3_ Doped MoO_2_ Precursor

MoO_2_ precursor with different La_2_O_3_ contents were synthesized via SCS using a mixture of AHM, La(NO_3_)_3_·6H_2_O, (NH_4_)NO_3_, and glycine as raw materials, as shown in Table 1, with different La_2_O_3_ content (0, 0.3, 0.6, 0.9, and 1.2 wt.%, i.e., the mass ratio of La_2_O_3_ and Mo), and labeled as P-LM0, P-LM3, P-LM6, P-LM9, and P-LM12, respectively. First, the mixture of metallic salts was dissolved in 100 mL of deionized water under magnetic stirring until a homogeneous solution was obtained. The solution was then poured into a 1000 mL beaker and heated at 160 °C in air using a temperature-controlled electric heating furnace. After heating for about 5 min at 160 °C, as soon as the solvent had evaporated, and a gelatinous mass was formed, an instantaneous combustion reaction occurred, accompanied by expansion of the gelatinous mass and the evolution of gas. The whole combustion reaction process lasted for less than 1 min and a foamy and black MoO_2_ precursor doped with different La_2_O_3_ contents, i.e., P-LM0, P-LM3, P-LM6, P-LM9, and P-LM12, was synthesized.

### 2.3. Hydrogen Reduction of Mo-Based Precursor

**Figure 1:**
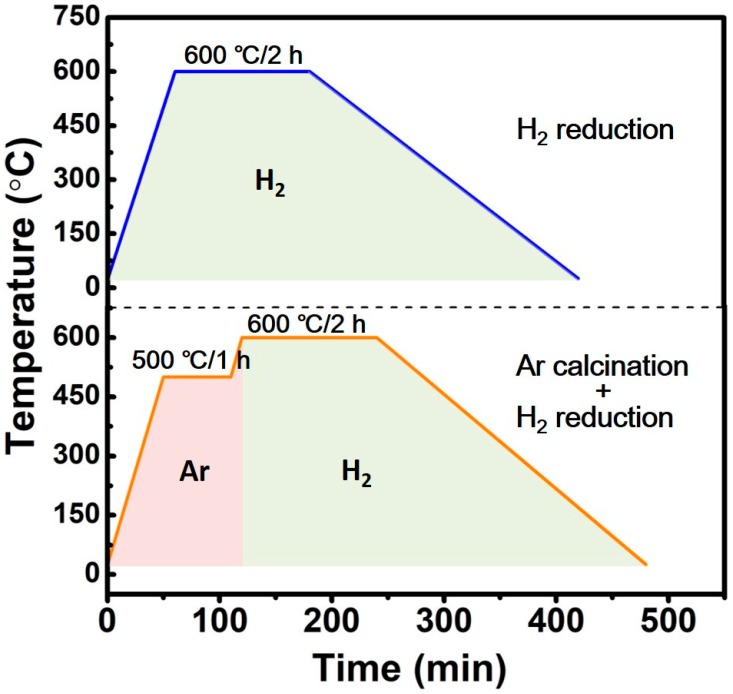
Direct H_2_ reduction process and Ar calcination-H_2_ reduction process for MoO_2_ precursors.

Lanthanum-based MoO_2_ precursors (P-LM0, P-LM3, P-LM6, P-LM9, and P-LM12) were reduced by using two different reduction processes, i.e., a direct H_2_ reduction process and an Ar calcination-H_2_ reduction process. The detailed reduction procedure is shown in Figure 1, and the reduced products are marked as LM0, LM3, LM6, LM9, and LM12, respectively.

### 2.4. Characterization

The crystalline phases of the samples were analyzed using an X-ray diffractometer (Rigaku, D/max-RB12, Tokyo, Japan) with Cu K*α* radiation, and a 2θ angle from 10° to 90° with 0.02° increments. The chemical composition at the surface was established using X-ray photoelectron spectroscopy (XPS, Fison VG ESCA210, West Sussex, England). The morphology and size distribution of the nanoparticles were investigated by field emission scanning electron microscopy (FE-SEM, Zeiss, ULTRA 55, Jena, Germany). Transmission electron microscopy (TEM) and high-resolution transmission electron microscopy (HRTEM) were performed on a transmission electron microscope (FEI, Tecnai G2 F20, Hillsboro, OR, USA) with an acceleration voltage of 200 kV. The carbon content in precursors and reduced samples was measured with a carbon/sulfur analyzer (NCS CS-2800, Beijing, China), and their La content was determined by inductively coupled plasma optical emission spectrometry (ICP-OES, Agilent 720, Santa Clara, CA, USA). The La_2_O_3_ content of the reduced samples was calculated based on the result of the La content.

## 3. Results and Discussion

### 3.1. Phases and Morphology of Precursor

**Figure 2:**
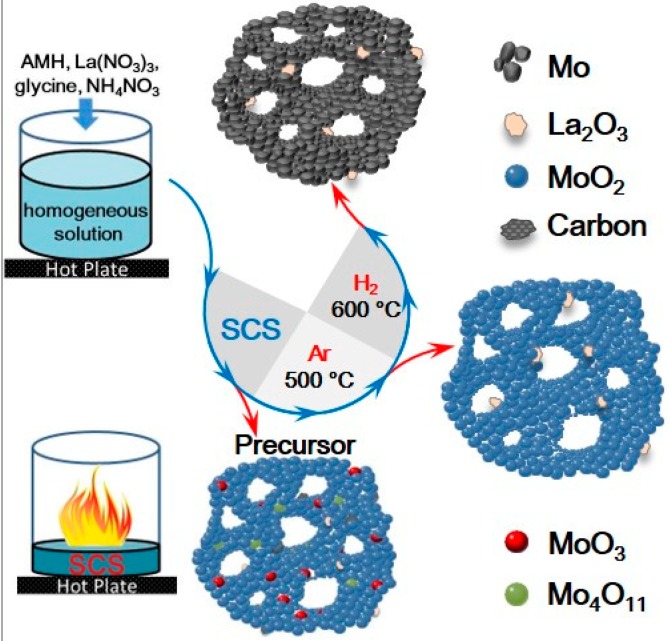
Schematic diagram describing the preparation of La_2_O_3_ uniformly doped Mo nanopowders via the SCSHR route.

A schematic diagram of the preparation of La_2_O_3_ uniformly doped Mo nanopowders via the SCSHR route is shown in Figure 2. First, MoO_2_ precursors with different La_2_O_3_ contents were synthesized via SCS using AHM, lanthanum nitrate, ammonium nitrate, and glycine as a molybdenum source, La source, oxidizer, and fuel, respectively. All raw materials were mixed and then heated at 160 °C in air. After about 5 min, an instantaneous combustion reaction occurred, which was accompanied by the evolution of a large amount of gas. The combustion process took less than 1 min and the target precursor samples were synthesized. Next, the obtained precursors were used to prepare La_2_O_3_ doped Mo nanopowders by a direct H_2_ reduction process and an Ar calcination-H_2_ reduction process, respectively. The detailed reduction procedure is shown in Figure 1.

**Figure 3:**
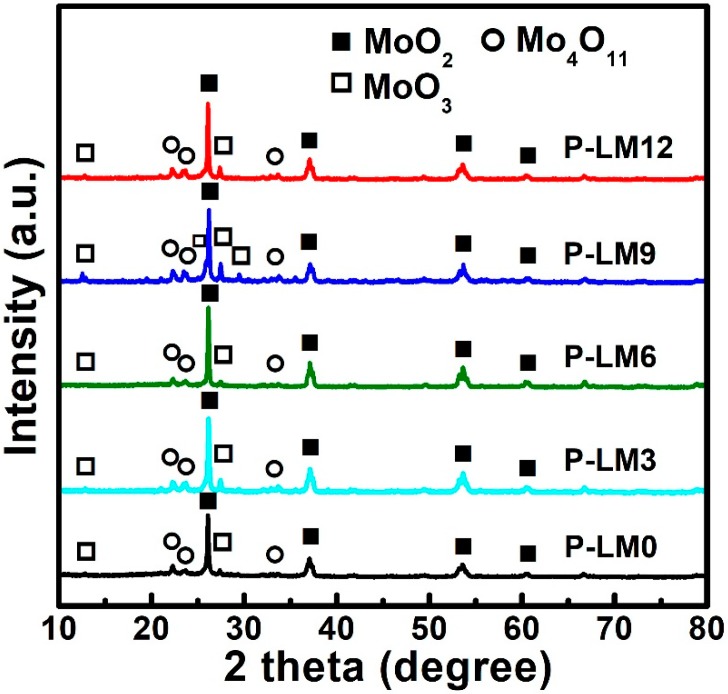
XRD patterns of MoO_2_ precursors doped with different La_2_O_3_ content and synthesized by SCS.

Figure 3 shows the XRD patterns of the different La_2_O_3_ doped precursors, synthesized by SCS. For all precursors, the diffractograms display the main diffraction peaks, which are indexed as monoclinic MoO_2_ (ICDD No. 78-1069). However, extremely small diffraction peaks corresponding to MoO_3_ and Mo_4_O_11_ phases are also observed in all XRD patterns, indicating that part of the MoO_2_ on the surface underwent oxidation in air. It was noted that both diffraction peaks of 26 and 29.5 two-theta degree appeared in the P-LM9 precursor sample, indicating that P-LM9 may contain more MoO_3_ phases than other samples. In addition, lanthanide compounds, such as La_2_O_3_ and La(OH)_3_ diffraction peaks were not observed for P-LM3, P-LM6, P-LM9, and P-LM12 samples, which was due to the low content of the La element. Nano-sized La_2_O_3_ has been synthesized by SCS in earlier studies [24,25,26]. Gangwar et al. [24] reported that a pure La_2_O_3_ phase can be synthesized by SCS followed by calcination treatment, while La_2_O_3_ converts to La(OH)_3_ upon exposure to the atmosphere due to its hygroscopic nature.

**Figure 4:**
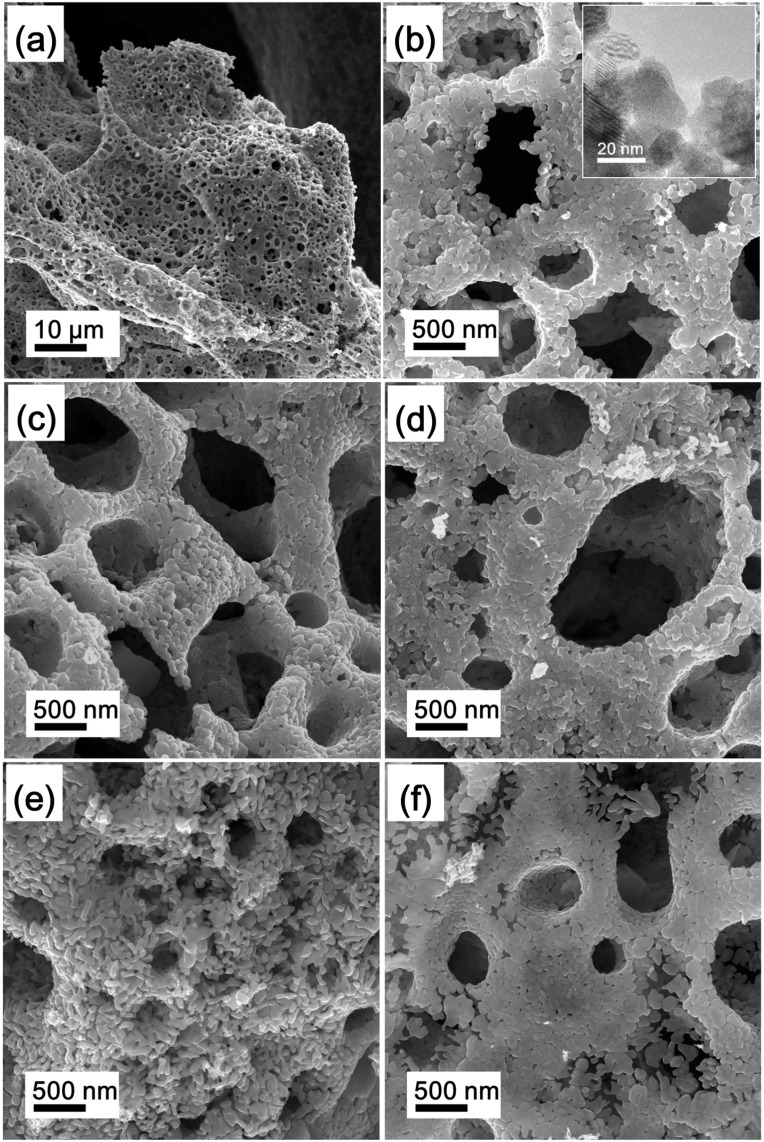
FE-SEM images of MoO_2_ precursors for (**a**,**b**) P-LM0, (**c**) P-LM3, (**d**) P-LM6, (**e**) P-LM9, and (**f**) P-LM12; the inset of (**b**) shows the corresponding TEM image.

The morphology and microstructure of the precursors were assessed by FE-SEM. Representative images are shown in Figure 4. It can be observed that the P-LM0 precursor sample has a foam-like morphology, as shown in Figure 4a, which is the unique typical morphology obtained by the SCS method. Figure 4b shows that the foam-like P-LM0 is composed of MoO_2_ nanoparticles with sizes of 20–40 nm as a result of particle agglomeration due to the high surface energy of MoO_2_ nanoparticles. Similarly, the morphology of La_2_O_3_ doped precursors, i.e., P-LM3, P-LM6, P-LM9, and P-LM12, still maintain the foam-like morphology and particle agglomeration. In addition, the particle size of La_2_O_3_ doped precursors does not change significantly, indicating that the morphology and particle size of precursors is not affected when doping with low La_2_O_3_ amounts, as shown in Figure 4c–f. However, due to the low loading degree of La_2_O_3_, the La_2_O_3_ nanoparticles are difficult to see in these images.

**Figure 5:**
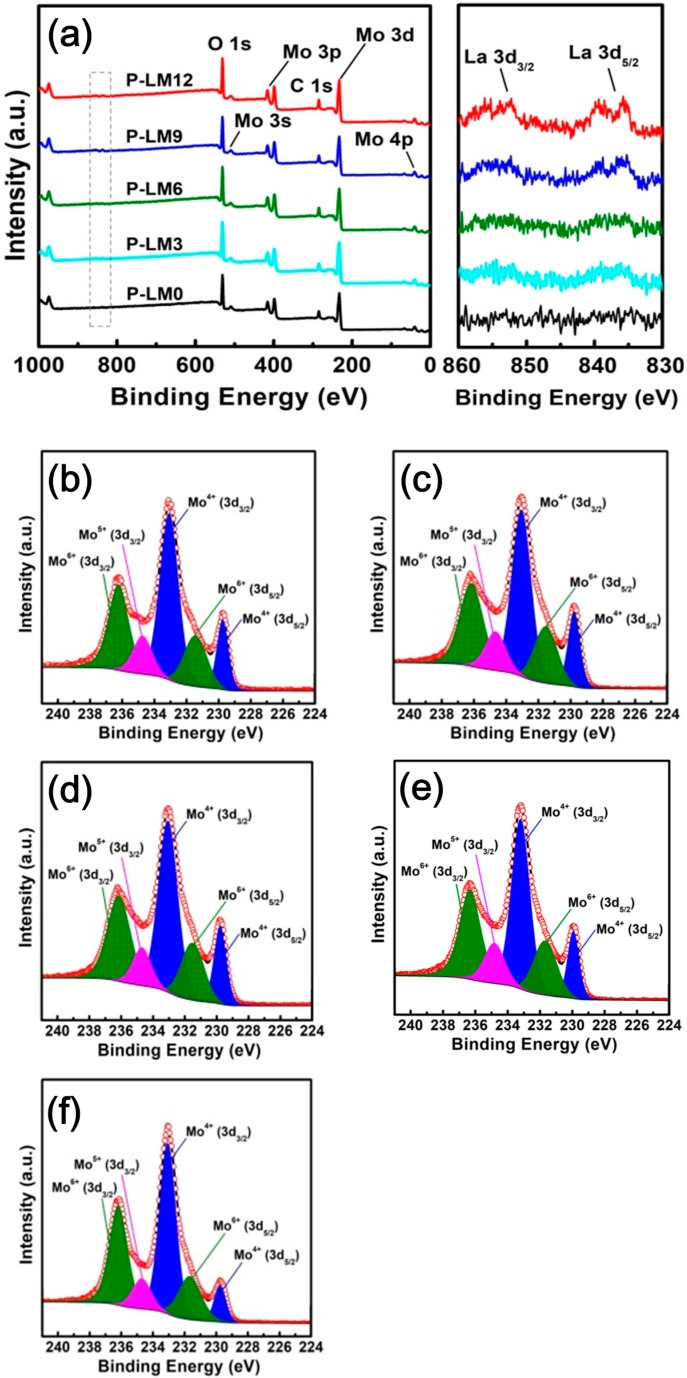
(**a**) XPS survey scan and La 3d spectra of each MoO_2_ precursor. Mo 3d high-resolution spectra of (**b**) P-LM0, (**c**) P-LM3, (**d**) P-LM6, (**e**) P-LM9, and (**f**) P-LM12.

To clarify whether La_2_O_3_ has been successfully doped into the precursor, XPS was performed. Figure 5 shows a survey scan, La 3d spectra, and Mo 3d high-resolution spectra for each precursor. As shown in Figure 5a, no peak of La 3d was observed in P-LM0. However, after La_2_O_3_ doping, peaks corresponding to La 3d_3/2_ and La 3d_5/2_ appeared whose intensities increase as the amount of La_2_O_3_ increases in the sample, clearly showing the successful doping of La_2_O_3_ into MoO_2_ precursors. The Mo 3d high-resolution spectra for each precursor display five peaks at 232.8 eV (Mo^4+^3d_3/2_ in MoO_2_), 299.6 eV (Mo^4+^3d_5/2_ in MoO_2_), 231.3 eV (Mo^6+^3d_5/2_ in MoO_3_), 235.9 eV (Mo^6+^3d_3/2_ in MoO_3_), and 234.4 eV (Mo^5+^3d_3/2_ in Mo_4_O_11_), as shown in Figure 5b–f. On the basis of these peaks, the contents of MoO_2_, MoO_3_, and Mo_4_O_11_ were calculated, and the results are listed in Table 2. As shown, the contents of impurities, such as MoO_3_ and Mo_4_O_11_, were found to be quite high, which far exceeds that indicated by the Rietveld refinement XRD result of a previous study by our research group [27]. This result can be related to the XPS technique, which can analyze only the surface with an information depth of up to 10 nm and can be affected by surface oxidation of the MoO_2_ nanoparticles. 

Table 3 shows the content of C and La elements in each precursor. As observed, each precursor contains traces of carbon. The La content is close to the theoretical content, indicating that the La element is not lost through the SCS method, which ensures the accuracy of La doping.

### 3.2. Preparation of La_2_O_3_ Doped Mo Nanopowders

As shown in Figure 1, the P-LM0, P-LM3, P-LM6, P-LM9, and P-LM precursors were reduced by both direct H_2_ reduction and Ar calcination-H_2_ reduction processes. The corresponding reduction products are referred to as LM0, LM3, LM6, LM9, and LM12 hereafter.

**Figure 6:**
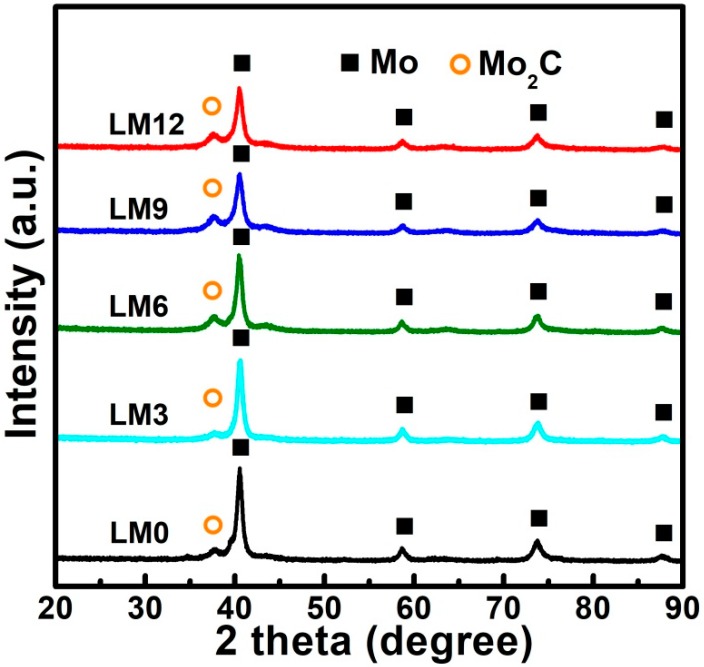
XRD patterns of products reduced by the direct H_2_ reduction process.

**Figure 7:**
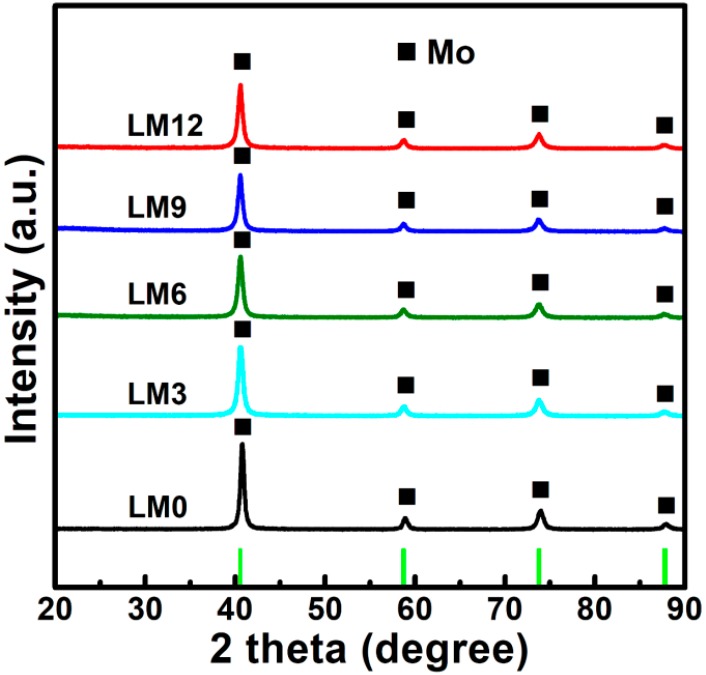
XRD patterns of products reduced by the Ar calcination-H_2_ reduction process.

The XRD patterns of the products reduced by direct H_2_ reduction and Ar calcination-H_2_ reduction process are illustrated in Figure 6 and Figure 7, respectively. As shown in Figure 6, the main diffraction peaks displayed by all diffractograms correspond to the Mo phase while no evidence for MoO_2_ peaks is found. This suggests that MoO_2_ and impurities (MoO_3_ and Mo_4_O_11_) were completely reduced to the metallic Mo phase. Yet, a small diffraction peak at around 38° can be observed in each diffraction pattern. It is attributed to the Mo_2_C phase, which may result from the reaction between residual carbon and MoO_2_ (or MoO_3_, Mo_4_O_11_). On the other hand, the XRD patterns of products obtained by the Ar calcination-H_2_ reduction process, as shown in Figure 7, shows that zero-valent Mo is the only crystalline phase while Mo_2_C does not appear anymore, clearly suggesting that the formation of Mo_2_C can be avoided via rational preparation of La_2_O_3_ doped Mo powders by the Ar calcination-H_2_ reduction process. Indeed, the C content was extremely low, i.e., 0.046, 0.043, 0.053, 0.051, and 0.042 wt.% for LM0, LM3, LM6, LM9, and LM12, respectively, as listed in Table 4. Moreover, the C content was much lower than that of samples obtained by direct H_2_ reduction. Therefore, in light of these results, it can be stated that the impurity-free La_2_O_3_ doped Mo powders can be successfully synthesized by the Ar calcination-H_2_ reduction process.

However, as mentioned above, the low amount of La_2_O_3_ did not allow its detection by XRD in any of the samples. Regarding Mo, the full width at half maxima (FWHM) of the peak was appropriate to calculate the crystallite size of Mo obtained by the Ar calcination-H_2_ reduction process by applying the Scherrer equation. Hence, crystallites of 28.7, 24.3, 23.8, 22.7, and 21.9 nm were obtained for LM0, LM3, LM6, LM9, and LM12, respectively.

**Figure 8:**
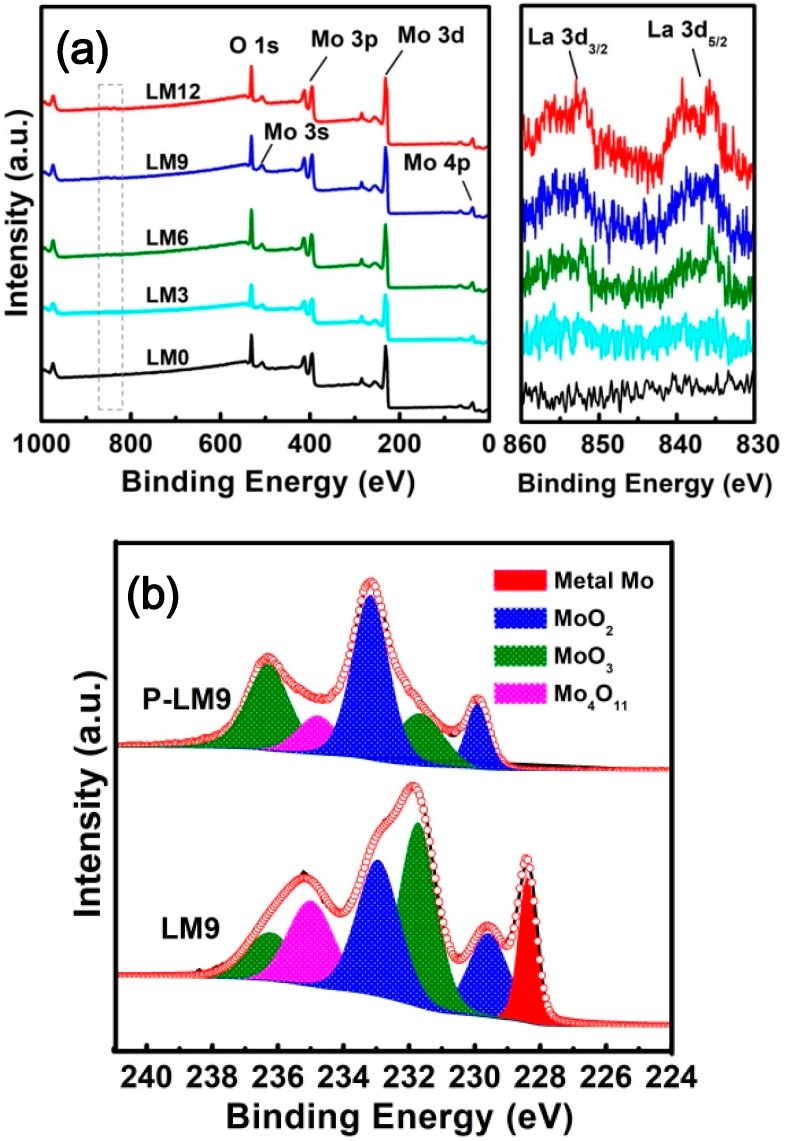
(**a**) XPS survey scan and La 3d spectra of La_2_O_3_ doped Mo nanopowders obtained by the Ar calcination-H_2_ reduction process, and (**b**) Mo 3d high-resolution spectra of P-LM9 and LM9.

Figure 8 shows the XPS survey scan and La 3d spectra of La_2_O_3_ doped Mo nanopowders obtained by the Ar calcination-H_2_ reduction process and Mo 3d high-resolution spectra for the P-LM9 and LM9 sample. As shown in Figure 8a, the increase in La_2_O_3_ doping resulted in more intense La 3d peaks, underlying the doping of Mo powders with La_2_O_3_. Figure 8b comparatively displays the Mo 3d high-resolution spectra of P-LM9 and LM9. It can be noticed that a new peak at 280.2 eV appeared in Mo 3d high-resolution spectra of LM9, which is ascribed to metallic Mo. Thus, this is evidence of the formation of a metallic Mo phase in the sample. In addition, well-shaped peaks corresponding to MoO_2_, MoO_3_, and Mo_4_O_11_ phases are observed for LM9, suggesting that Mo nanoparticles at the surface were oxidized in air. The Mo 3d high-resolution spectra for the other samples, i.e., LM0, LM3, LM6, and LM12, show similar characteristics to that of LM9.

ICP-OES was used to analyze the La content in La_2_O_3_ doped Mo nanopowders reduced by the Ar calcination-H_2_ reduction process. Accordingly, the La_2_O_3_ contents as calculated from La content of LM3, LM6, LM9, and LM12 samples are 0.27, 0.64, 0.88, and 1.16 wt.%, respectively. These values are close to the theoretical values, showing that the synthetic approach used herein to prepare La_2_O_3_ doped Mo powders is suitable for a complete incorporation of the doping element.

**Figure 9:**
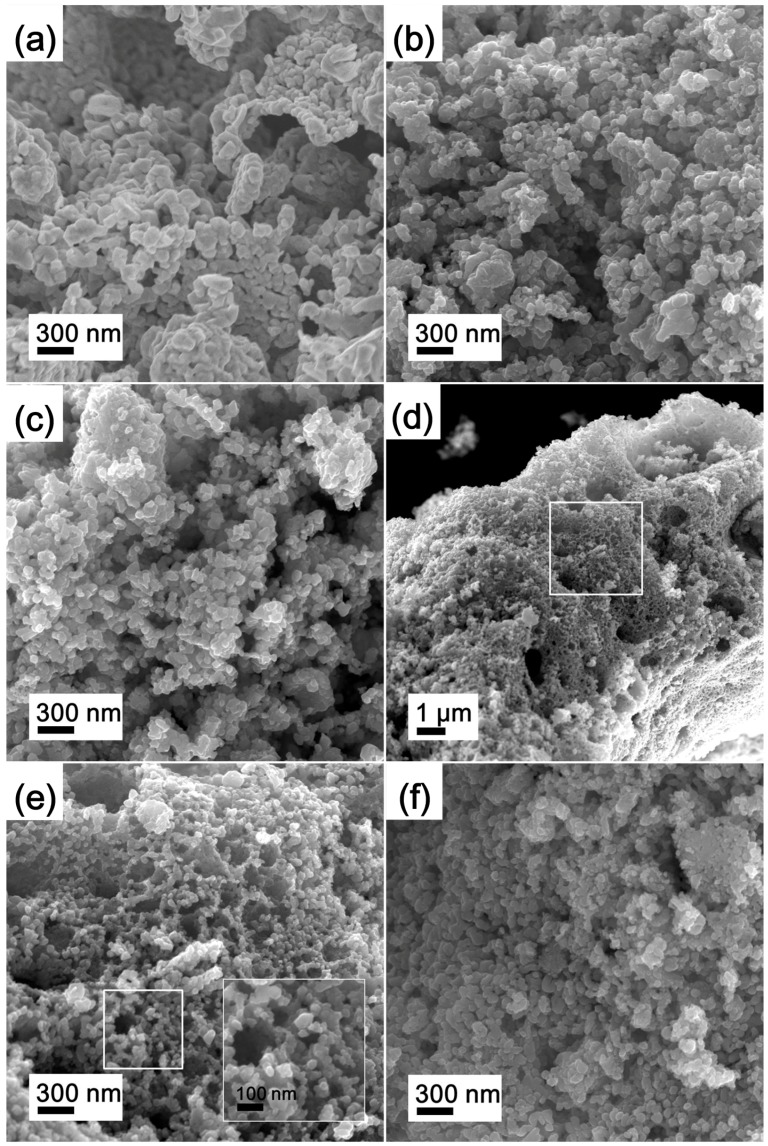
FE-SEM images of La_2_O_3_ doped Mo nanopowders for (**a**) LM0, (**b**) LM3, (**c**) LM6, (**d**,**e**) LM9, and (**f**) LM12 reduced by the Ar calcination-H_2_ reduction process.

FE-SEM images of La_2_O_3_ doped Mo nanopowders reduced by the Ar calcination-H_2_ reduction process are displayed in Figure 9. Aggregates composed of nanoparticles are clearly seen for each product. The foam-like morphology seen in Figure 9d indicates that the samples maintain the original morphology of MoO_2_ precursor during the reduction process. The average particle sizes, obtained from the SEM images, increase as the amount of La_2_O_3_ decreases (43 nm (LM12) > 49 nm (LM9) > 58 nm (LM6) > 63 nm (LM3) > 68 nm (LM0)), revealing the inhibition of the segregation process induced by La_2_O_3_ doping.

**Figure 10:**
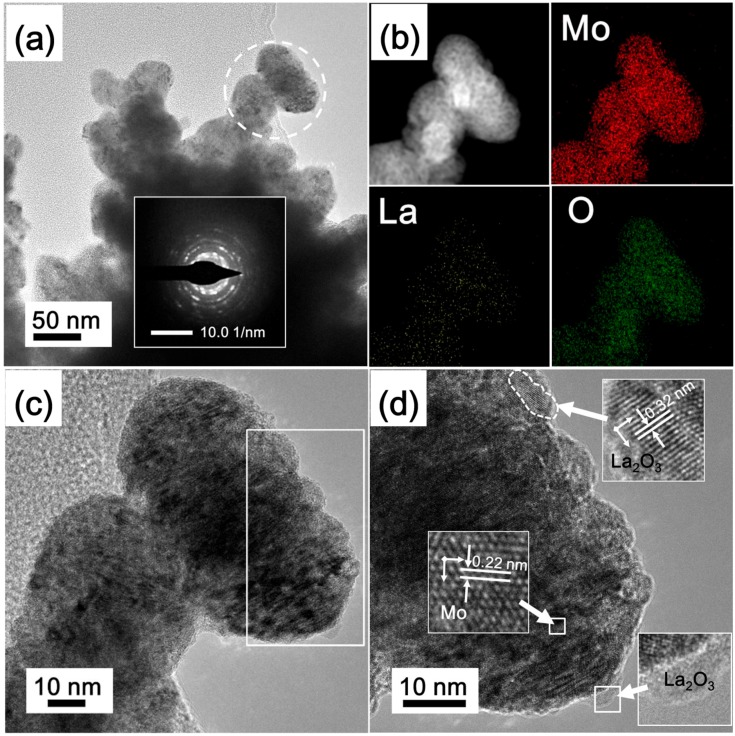
(**a**) TEM, (**b**) elemental mapping, (**c**,**d**) HRTEM images of La_2_O_3_ doped Mo nanopowders (LM9 sample); the inset of (**a**) shows the corresponding SAED pattern; the insets of (**d**) show the lattice fringes of metallic Mo and La_2_O_3_.

TEM micrographs of the LM9 product reduced by the Ar calcination-H_2_ reduction process are shown in Figure 10. Figure 10a displays aggregates of irregular shapes of Mo nanoparticles. The corresponding selected-area diffraction electron patterns (SAED), shown in the inset, exhibit a polycrystalline ring pattern, indicating that the aggregate consists of Mo nanoparticles, which agrees with the FE-SEM observations. The elemental mapping images, shown in Figure 10b, demonstrate the uniform distribution of Mo, La, and O elements throughout the particles, and confirm the uniform La_2_O_3_ doping into Mo nanopowders via the SCSHR route. It is worth mentioning that the relatively high content of O observed in the O element mapping image may be explained by (i) its origin in La_2_O_3_ and (ii) the oxidation of surface Mo nanoparticles. 

Figure 10c,d shows the HRTEM images taken from the selected area in Figure 10a. Two different lattice fringes are clearly observed. Rich lattice fringes with spacing of 0.22 nm correspond to the (110) plane of metallic Mo. Other small lattice fringes are clearly seen at the edge of particles while lattice spacing of 0.32 nm, which agrees well with the (222) crystalline plane of La_2_O_3_. This result further confirms the successful doping of La_2_O_3_ into Mo nanopowders.

### 3.3. Formation Mechanism of La_2_O_3_ Doped Mo Nanopowders

According to theory, SCS involves strong oxidation–reduction reactions. In this reaction system, the La_2_O_3_ doped MoO_2_ precursors were formed by Equation (1) and Equation (2). Furthermore, a large amount of gases generated led to the formation of foam-like morphology [23]. Because the SCS method involves reactions of precursors in solution, the incorporation of the entire amount of doping element is ensured, as well as the uniform distribution of La_2_O_3_ into the host material.

(1) $$\;{\left({\mathrm{NH}}_{4}\right)}_{6}{\mathrm{Mo}}_{7}O_{24}+\;20{\;\mathrm{NH}}_{4}{\mathrm{NO}}_{3}+10{\;C}_{2}H_{5}O_{2}N\;\to 7{\;\mathrm{MoO}}_{2}+32{\;H}_{2}O+30{\;\mathrm{NH}}_{3}+\;20{\;\mathrm{CO}}_{2}+18\;\mathrm{NO}+4{\;N}_{2}\;$$

(2) $$\;6\;\mathrm{La}{\left({\mathrm{NO}}_{3}\right)}_{3}\;+10{\;C}_{2}H_{5}O_{2}N\to 3{\;\mathrm{La}}_{2}O_{3}+25{\;H}_{2}O+20{\;\mathrm{CO}}_{2}+14{\;N}_{2}\;$$

Recent studies have shown that the reduction mechanism of MoO_2_ to Mo under H_2_ depends on the experimental conditions, in particular, the temperature of reduction [28]. The reduction reaction obeys the chemical vapor transport (CVT) mechanism, when the reaction temperature is above 923 K (650 °C). When the reaction temperature is lower than 883 K (610 °C), the mechanism is a pseudomorphic transport mechanism, which results in the nucleation and growth of Mo inside MoO_2_ particles. Thus, the Mo products will retain the morphology and size of the initial MoO_2_ particles. In light of these results, the preservation of the foam-like morphology and nanostructure of the MoO_2_ precursor by the La_2_O_3_ doped Mo products obtained at 600 °C is explained.

When the precursors were prepared by the direct H_2_ reduction process, the impurities consisting of MoO_3_ and Mo_4_O_11_ were reduced to MoO_2_ based on Equations (3) and (4) [29], respectively. At 600 °C, Equation (5) takes place, forming metallic Mo nanoparticles. Furthermore, an insignificant amount of Mo_2_C impurity formed as a result of the carbothermal reduction in hydrogen of MoO_2_ [27,30,31], as shown by Equation (6). La_2_O_3_ does not undergo any phase transition due to its high thermodynamic stability.

(3) $$\;{\mathrm{MoO}}_{3}\;+H_{2}\to {\mathrm{MoO}}_{2}+H_{2}O\;$$

(4) $$\;{\mathrm{Mo}}_{4}O_{11}\;+3{\;H}_{2}\to 4{\;\mathrm{MoO}}_{2}+3{\;H}_{2}O\;$$

(5) $$\;{\mathrm{MoO}}_{2}\;+2{\;H}_{2}\to \mathrm{Mo}+2{\;H}_{2}O\;$$

(6) $$\;2{\;\mathrm{MoO}}_{2}\;+3\;C\;+2{\;H}_{2}\to {\mathrm{Mo}}_{2}C+2{\;H}_{2}O+2\;\mathrm{CO}\;$$

(7) $$\;2{\;\mathrm{MoO}}_{3}\;+C\to 2{\;\mathrm{MoO}}_{2}+{\mathrm{CO}}_{2}\;$$

(8) $$\;{\mathrm{Mo}}_{4}O_{11}\;+3\;C\to 4{\;\mathrm{MoO}}_{2}+3\;\mathrm{CO}\;$$

When the precursors were calcined in Ar at 500 °C, residual carbon was completely consumed via carbothermal reduction, i.e., Equations (7) and (8). Therefore, the La_2_O_3_ doped Mo nanopowders without Mo_2_C impurity were formed via the Ar calcination-H_2_ reduction process.

## 4. Conclusions

Mo nanopowders doped with different amounts of La_2_O_3_, i.e., 0, 0.27, 0.64, 0.88, and 1.16 wt.%, were prepared via the SCSHR route, which allowed a homogeneous distribution of the dopant while preserving the theoretical loading degree. The MoO_2_ precursors with different content of La_2_O_3_, synthesized by SCS, displayed similar foam-like morphology and particle sizes of 20–40 nm. Then, La_2_O_3_ uniformly doped Mo nanopowders were prepared via different reduction processes, i.e., the direct H_2_ reduction and Ar calcination-H_2_ reduction. Compared to the direct H_2_ reduction processes, the Ar calcination-H_2_ reduction process led to La_2_O_3_ uniformly doped Mo nanopowders without Mo_2_C impurities. La_2_O_3_ uniformly doped Mo nanopowders were composed of nanoparticles with sizes of 40–70 nm, which was in line with the decrease in La_2_O_3_. Hence, La_2_O_3_ doped Mo nanopowders retained the original morphology and size of the MoO_2_ precursor because the formation of Mo nanoparticles reduced at 600 °C obeys the pseudomorphic transport mechanism. The formation of Mo_2_C impurity was noticed due to the carbothermal reduction occurring when samples were directly reduced under H_2_. On the other hand, when the Ar calcination-H_2_ reduction process was applied to form lanthana-based materials, the residual carbon was completely consumed via the carbothermal reduction during the calcination in Ar at 500 °C and thus La_2_O_3_ doped Mo nanopowders without Mo_2_C impurity can be successfully prepared. The results obtained herein are valuable to recommend the SCSHR method for preparation of other oxide doped refractory metal nanopowders (e.g., tungsten and niobium).

## Figures and Tables

**Table 1:** Amount of raw materials used for the solution combustion synthesis (SCS) of La_2_O_3_ doped MoO_2_ precursors.

Precursor	La_2_O_3_ Doping Content (wt.%)	AHM (mol)	NH_4_NO_3_ (mol)	C_2_H_5_O_2_N (mol)	La(NO_3_)_3_ (mol)
P-LM0	0	0.01	0.2	0.10	0
P-LM3	0.3	0.01	0.2	0.10	0.00012
P-LM6	0.6	0.01	0.2	0.10	0.00025
P-LM9	0.9	0.01	0.2	0.10	0.00037
P-LM12	1.2	0.01	0.2	0.10	0.00050

**Table 2:** Contents of MoO_2_, MoO_3_, and Mo_4_O_11_ in each MoO_2_ precursor calculated based on the XPS peak area for Mo 3d.

Precursor	MoO_2_ (wt.%)	MoO_3_ (wt.%)	Mo_4_O_11_ (wt.%)
P-LM0	52.6	38.8	8.6
P-LM3	51.7	38.7	9.6
P-LM6	51.0	39.1	9.9
P-LM9	48.9	41.6	9.3
P-LM12	48.6	42.8	8.6

**Table 3:** Content of C and La element in each MoO_2_ precursor.

Precursors	P-LM0	P-LM3	P-LM6	P-LM9	P-LM12
Elements C (wt.%)	0.27	0.17	0.20	0.18	0.13
Elements La (wt.%)	0	0.13	0.38	0.59	0.81

**Table 4:** The content of C in the products reduced through different reduction processes.

Content	Process	Samples				
		LM0	LM3	LM6	LM9	LM12
C (wt.%)	H_2_/600 °C	0.286	0.219	0.241	0.236	0.183
	Ar/500 °C + H_2_/600 °C	0.046	0.043	0.053	0.051	0.042

## References

[1] Perepezko J.H. (2009). The hotter the engine, the better. Science.

[2] Liu G., Zhang G.J., Jiang F., Ding X.D., Sun Y.J., Sun J., Ma E. (2013). Nanostructured high-strength molybdenum alloys with unprecedented tensile ductility. Nat. Mater..

[3] Lenchuk O., Rohrer J., Albe K. (2016). Atomistic modelling of zirconium and silicon segregation at twist and tilt grain boundaries in molybdenum. J. Mater. Sci..

[4] Zhou Y., Gao Y., Wei S., Pan K., Hu Y. (2016). Preparation and characterization of Mo/Al_2_O_3_ composites. Int. J. Refract. Met. Hard Mater..

[5] El-Genk M.S., Tournier J.M. (2005). A review of refractory metal alloys and mechanically alloyed-oxide dispersion strengthened steels for space nuclear power systems. J. Nucl. Mater..

[6] Conduit B.D., Jones N.G., Stone H.J., Conduit G.J. (2018). Probabilistic design of a molybdenum-base alloy using a neural network. Scr. Mater..

[7] Wang K.S., Tan J.F., Hu P., Yu Z.T., Yang F., Hu B.L., Song R., He H.C., Volinsky A.A. (2015). La_2_O_3_ effects on TZM alloy recovery, recrystallization and mechanical properties. Mater. Sci. Eng. A.

[8] Cheng P.M., Zhang G.J., Zhang J.Y., Liu G., Sun J. (2015). Coupling effect of intergranular and intragranular particles on ductile fracture of Mo-La_2_O_3_ alloys. Mater. Sci. Eng. A.

[9] Wang L., Liu G., Sun J. (2017). Effects of La_2_O_3_ and annealing temperature on grain size and mechanical properties of Mo alloys. Mater. Res. Express.

[10] Yang X., Tan H., Lin N., Li Z., He Y. (2015). The influences of La doping method on the microstructure and mechanical properties of Mo alloys. Int. J. Refract. Met. Hard Mater..

[11] Yang X., Tan H., Lin N., Li Z., He Y. (2016). Effects of the lanthanum content on the microstructure and properties of the molybdenum alloy. Int. J. Refract. Met. Hard Mater..

[12] Zhang G.J., Sun Y.J., Zuo C., Wei J.F., Sun J. (2008). Microstructure and mechanical properties of multi-components rare earth oxide-doped molybdenum alloys. Mater. Sci. Eng. A.

[13] Cockeram B.V. (2009). The fracture toughness and toughening mechanism of commercially available unalloyed molybdenum and oxide dispersion strengthened molybdenum with an equiaxed, large grain structure. Metall. Mater. Trans. A.

[14] Chen C., Wang S., Jia Y.L., Wang M.P., Li Z., Wang Z.X. (2014). The microstructure and texture of Mo-La_2_O_3_ alloys with high transverse ductility. J. Alloy Compd..

[15] Endo M., Kimura K., Udagawa T., Tanabe S., Seto H. (1990). The effects of doping molybdenum wire with rare-earth elements. High Temp. High Press..

[16] Zhang J., Liu L., Zhou M., Hu Y., Zuo T. (1999). Fracture toughness of sintered Mo-La_2_O_3_ alloy and the toughening mechanism. Int. J. Refract. Met. Hard Mater..

[17] Chen P., Qin M., Zhang D., Chen Z., Jia B., Wan Q., Wu H., Qu X. (2015). Combustion synthesis and excellent photocatalytic degradation properties of W_18_O_49_. CrystEngComm.

[18] Huang M., Qin M., Chen P., Jia B., Chen Z., Li R., Liu Z., Qu X. (2016). Facile preparation of network-like porous hematite (α-Fe_2_O_3_) nanosheets via a novel combustion-based route. Ceram. Int..

[19] Bakrania S.D., Miller T.A., Perez C., Wooldridge M.S. (2007). Combustion of multiphase reactants for the synthesis of nanocomposite materials. Combust. Flame.

[20] Wu H., Qin M., Chu A., Cao Z., Chen P., Liu Y., Qu X. (2016). Effect of urea on the synthesis of Al-doped ZnO nanoparticle and its adsorptive properties for organic pollutants. Mater. Res. Bull..

[21] Cao Z., Qin M., Gu Y., Jia B., Chen P., Qu X. (2016). Synthesis and characterization of Sn-doped hematite as visible light photocatalyst. Mater. Res. Bull..

[22] Chen P., Qin M., Liu Y., Jia B., Cao Z., Wan Q., Qu X. (2015). Superior optical properties of Fe^3+^–W_18_O_49_ nanoparticles prepared by solution combustion synthesis. New J. Chem..

[23] Gu S., Qin M., Zhang H., Ma J., Wu H., Qu X. (2017). Facile solution combustion synthesis of MoO_2_ nanoparticles as efficient photocatalysts. CrystEngComm.

[24] Gangwar B.P., Palakollu V., Singh A., Kanvah S., Sharma S. (2014). Combustion synthesized La_2_O_3_ and La(OH)_3_: recyclable catalytic activity towards Knoevenagel and Hantzsch reactions. RSC Adv..

[25] Singh A., Palakollu V., Pandey A., Kanvah S., Sharma S. (2016). Green synthesis of 1,4-benzodiazepines over La_2_O_3_ and La(OH)_3_ catalysts: possibility of Langmuir–Hinshelwood adsorption. RSC Adv..

[26] Nowicki W., Piskuła Z.S., Kuźma P., Kirszensztejn P. (2017). Synthesis and characterization of a binary system La_2_O_3_–SiO_2_ prepared by combustion method. J. Sol Gel Sci. Technol..

[27] Gu S., Qin M., Zhang H., Ma J., Qu X. (2018). Preparation of Mo nanopowders through hydrogen reduction of a combustion synthesized foam-like MoO_2_ precursor. Int. J. Refract. Met. Hard Mater..

[28] Wang L., Zhang G.H., Wang J.S., Chou K.C. (2016). Study on hydrogen reduction of ultrafine MoO_2_ to produce ultrafine Mo. J. Phys. Chem. C.

[29] Ressler T., Jentoft R.E., Wienold J., Günter M.M., Timpe O. (2000). In stu XAS and XRD studies on the formation of Mo suboxides during reduction of MoO_3_. J. Phys. Chem. B.

[30] Liang C., Ying P., Li C. (2002). Nanostructured β-Mo_2_C prepared by carbothermal hydrogen reduction on ultrahigh surface area carbon material. Chem. Mater..

[31] Yang Y., Luo M., Xing Y., Wang S., Zhang W., Lv F., Li Y., Zhang Y., Wang W., Guo S. (2018). A Universal strategy for intimately coupled carbon nanosheets/MoM nanocrystals (M = P, S, C, and O) hierarchical hollow nanospheres for hydrogen evolution catalysis and sodium-ion storage. Adv. Mater..

